# Machine Perfusion in Liver Transplantation: A Systematic Review and Meta-Analysis Comparing Outcomes with Conventional Static Cold Storage

**DOI:** 10.3390/jcm15155950

**Published:** 2026-07-30

**Authors:** Jamilya Saparbay, Timur Lesbekov, Yuliya Semenova, Zhandos Burkitbayev

**Affiliations:** 1 Department of Surgery, Nazarbayev University School of Medicine, 5/1 Kerey and Zhanibek Khans Street, Astana 010000, Kazakhstan; yuliya.semenova@nu.edu.kz; 2 Hepatobiliary and Transplant Surgery, National Research Oncology Center, Astana 010000, Kazakhstan; zhan11@mail.ru; 3 Department of Cardiosurgery, University Medical Center—Heart Center, Turan Avenue 38, Astana 010000, Kazakhstan; lesbekovt@gmail.com

**Keywords:** liver transplantation, machine perfusion, static cold storage, hypothermic machine perfusion, normothermic machine perfusion, meta-analysis, systematic review, early allograft dysfunction, graft survival

## Abstract

**Background:** Static cold storage (SCS) remains the conventional standard for liver graft preservation; however, ischemia–reperfusion injury during storage may contribute to graft dysfunction, particularly with extended criteria and marginal donors. Machine perfusion (MP) has emerged as a promising alternative, yet existing systematic reviews are limited by their focus on individual perfusion modalities or selected donor populations. We performed a comprehensive systematic review and meta-analysis with pre-specified modality-specific subgroup analyses comparing all MP strategies with SCS in adult liver transplantation. **Methods:** This study was conducted in accordance with PRISMA 2020 guidelines and registered in PROSPERO (CRD420261355605). MEDLINE, Embase, and PubMed were systematically searched for comparative studies published between 2015 and 2025. Primary outcomes were early allograft dysfunction (EAD), primary non-function (PNF), and 1-year graft survival. Secondary outcomes included biliary complications, hepatic artery thrombosis (HAT), and postoperative transaminase levels. Pre-specified subgroup analyses were performed according to perfusion modality (NMP versus HMP/HOPE). **Results:** A total of 1448 records were identified. After removal of 309 duplicates and screening, 21 studies comprising 3665 patients were included. Compared with SCS, MP was associated with a significantly lower risk of EAD (RR 0.67, 95% CI 0.48–0.94; *p* = 0.020), PNF (RR 0.31, 95% CI 0.11–0.84; *p* = 0.020), and graft loss at one year (RR 0.54, 95% CI 0.36–0.81; *p* = 0.003; I^2^ = 0%). Subgroup analysis demonstrated that reductions in EAD and PNF were driven by HMP/HOPE strategies (EAD: RR 0.66, 95% CI 0.45–0.98; PNF: RR 0.23, 95% CI 0.07–0.79), whereas NMP did not achieve statistical significance for these endpoints. No significant differences were observed in biliary complications or HAT. **Conclusions:** Machine perfusion is associated with improved early graft function, reduced primary non-function, and superior 1-year graft survival compared with static cold storage. The benefit for early graft outcomes is driven by hypothermic strategies. These findings provide modality-specific evidence to guide preservation strategy selection in clinical practice.

## 1. Introduction

Liver transplantation remains the most effective treatment for end-stage liver disease, providing life-saving therapy for thousands of patients annually [1]. One of the principal limitations of transplantation is the persistent gap between organ supply and recipient demand [2,3], which has driven increasing utilization of extended criteria donor (ECD) grafts and organs recovered after circulatory death. These marginal grafts are inherently more susceptible to preservation-related injury, making the choice of preservation strategy critically important.

Static cold storage (SCS) remains the conventional standard for liver graft preservation due to its simplicity, low cost, and broad applicability. By reducing cellular metabolism and oxygen demand, SCS extends tolerable ischemia time; however, metabolic activity is not entirely halted during cold storage, and ischemia–reperfusion injury remains an inevitable consequence of the transplantation process [4,5]. SCS has inherent biological limitations, including cellular swelling, acidosis, and accumulation of reactive oxygen species, which are particularly detrimental in ECD grafts that exhibit impaired microcirculation and reduced tolerance to oxidative stress [5].

Machine perfusion (MP) has emerged as a biologically plausible and increasingly adopted alternative to SCS. Initially developed in cardiac surgery, it has been subsequently introduced into liver transplantation. Three principal strategies have been developed: hypothermic machine perfusion (HMP), hypothermic oxygenated perfusion (HOPE), and normothermic machine perfusion (NMP), differing in temperature, perfusate composition, and degree of oxygenation [6]. Unlike SCS, machine perfusion maintains continuous flow through the graft, facilitates mitochondrial recovery, and in the case of normothermic approaches, allows real-time functional assessment of the graft prior to implantation.

While numerous studies have reported benefits of machine perfusion over static cold storage, the published evidence remains heterogeneous and inconsistent. Importantly, existing systematic reviews have predominantly focused on individual perfusion modalities—either hypothermic or normothermic strategies—or have examined selected donor populations such as donation after circulatory death grafts alone. Many do not provide quantitative synthesis of the most clinically relevant outcomes, including EAD, PNF, and graft survival, across the full spectrum of machine perfusion strategies. Furthermore, several landmark randomized controlled trials published between 2023 and 2025 have not yet been incorporated into a comprehensive pooled analysis.

The aim of this systematic review and meta-analysis was therefore to provide an updated and comprehensive synthesis of the current evidence comparing all machine perfusion strategies with static cold storage in adult liver transplantation. Crucially, we performed pre-specified subgroup analyses by perfusion modality—separating HMP/HOPE from NMP—to determine whether the benefits of machine perfusion differ according to the biological mechanism of preservation employed. This modality-specific approach addresses a key methodological gap in prior meta-analyses and provides more actionable guidance for clinical practice.

## 2. Methods

Study Design and Registration

This systematic review and meta-analysis was conducted in accordance with the Preferred Reporting Items for Systematic Reviews and Meta-Analyses (PRISMA) 2020 guidelines and was prospectively registered in PROSPERO (CRD420261355605).

Search Strategy

A comprehensive literature search was conducted across MEDLINE, Embase, and PubMed from January 2015 to December 2025. The search strategy combined the following terms: (“liver transplant” OR “liver transplantation” OR “hepatic transplantation” OR “orthotopic transplantation”) AND (“machine perfusion” OR “normothermic machine perfusion” OR “hypothermic machine perfusion” OR “ex situ perfusion” OR “dual hypothermic oxygenated perfusion”) AND (“static cold storage” OR “cold storage” OR “organ preservation”). Reference lists of retrieved articles were manually screened for additional eligible studies.

Eligibility Criteria

Studies were included if they met the following criteria: (1) adult liver transplant recipients (age ≥ 18 years); (2) use of any machine perfusion strategy applied to the graft prior to implantation; (3) comparison with static cold storage using standard preservation solutions; and (4) reporting at least one pre-specified outcome of interest. Both randomized controlled trials and observational comparative studies (including prospective and retrospective cohort and case–control studies) were eligible.

Studies were excluded if they: included only pediatric populations; lacked a comparator group; were non-comparative case series; represented animal or experimental studies without clinical transplantation data; or were conference abstracts, editorials, letters, case reports, dissertations, or unpublished studies with insufficient methodological detail.

Outcomes

Primary outcomes were: (1) early allograft dysfunction (EAD), defined according to the Olthoff criteria (bilirubin ≥ 10 mg/dL on day 7, INR ≥ 1.6 on day 7, or ALT/AST > 2000 IU/L within the first 7 days); (2) primary non-function (PNF); and (3) 1-year graft survival. Secondary outcomes included biliary complications, hepatic artery thrombosis (HAT), and postoperative liver injury markers (peak ALT and AST).

Baseline donor and recipient characteristics, including age, Model for End-Stage Liver Disease (MELD) score, donor type (DBD/DCD), and perfusion modality, were extracted descriptively and were not included in pooled meta-analysis.

Data Extraction and Risk of Bias Assessment

Data were independently extracted by two reviewers using a standardized extraction form. Discrepancies were resolved by discussion and consensus. Risk of bias in randomized controlled trials was assessed using the Cochrane Risk of Bias 2 (RoB 2) tool, which evaluates five domains: randomization process, deviations from intended interventions, missing outcome data, outcome measurement, and selection of reported results. Observational studies were assessed using the ROBINS-I tool across seven domains, with the Newcastle-Ottawa Scale (NOS) applied where ROBINS-I was not feasible due to incomplete reporting. Studies assessed as “some concerns” (RoB 2) or “moderate” (ROBINS-I) were categorized as moderate risk of bias for consistency.

Statistical Analysis

All statistical analyses were performed using Stata software (version 18, StataCorp, College Station, TX, USA). Pooled effect estimates were calculated using a random-effects model (DerSimonian–Laird method) to account for anticipated clinical and methodological heterogeneity.

For dichotomous outcomes (EAD, PNF, biliary complications, HAT, and 1-year graft survival), pooled risk ratios (RRs) with 95% confidence intervals (CIs) were calculated. For continuous outcomes (peak AST and ALT), pooled standardized mean differences (SMDs) with 95% CIs were used owing to variability in units and reporting formats across studies.

Statistical heterogeneity was assessed using Cochran’s Q test and quantified using the I^2^ statistic, with thresholds defined as: low (<25%), moderate (25–50%), substantial (50–75%), and considerable (>75%). Sensitivity analyses used a leave-one-out approach to evaluate the robustness of pooled estimates; additionally, analyses excluding studies with extreme variance were conducted for transaminase outcomes. Pre-specified subgroup analyses according to perfusion modality (NMP versus HMP/HOPE) were performed for all primary outcomes, with between-subgroup differences assessed using a test for interaction. A *p*-value < 0.05 was considered statistically significant.

AI assistance Disclosure

During the preparation of this manuscript, the authors used Claude (Anthropic, San Francisco, CA, USA) to assist with manuscript editing and language refinement, and to redraw forest plot figures at publication-quality resolution from author-verified data. The authors have reviewed and verified all AI-assisted content.

## 3. Results

Study Selection and Characteristics

**Figure 1:**
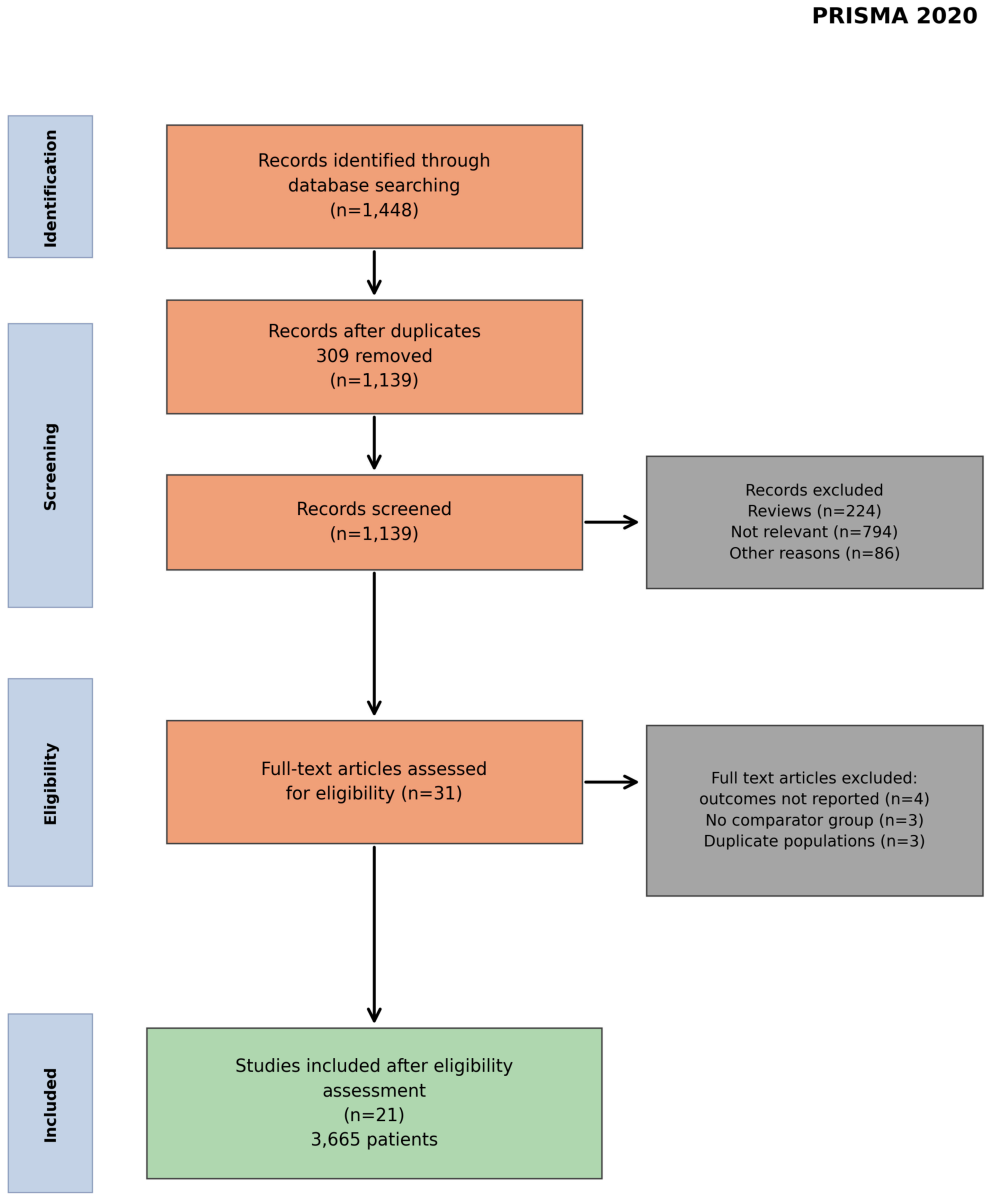
PRISMA 2020 flow diagram of study selection.

The database search identified 1448 records. After removal of 309 duplicates, 1139 records were screened based on titles and abstracts. A total of 1104 records were excluded (224 review articles, 794 not relevant to the research question, and 86 excluded for other reasons). Thirty-one full-text articles were assessed for eligibility, of which 10 were excluded for the following reasons: outcomes not reported in extractable format (*n* = 4), absence of a static cold storage comparator group (*n* = 3), and duplicate or overlapping study populations (*n* = 3). A total of 21 studies were included in the final meta-analysis [7,8,9,10,11,12,13,14,15,16,17,18,19,20,21,22,23,24,25,26,27]. The PRISMA 2020 flow diagram is presented in Figure 1, and the completed PRISMA 2020 checklist is provided in the Supplementary Materials.

The characteristics of the included studies are summarized in Table 1. The 21 studies comprised a total of 3665 patients. Studies were conducted across Europe (*n* = 14), North America (*n* = 5), Asia (*n* = 1), and multi-country collaborations (*n* = 1), and were published between 2015 and 2025. Machine perfusion modalities included NMP (*n* = 9) and hypothermic strategies comprising HMP, HOPE, and dual HOPE (dHOPE) (*n* = 12). Six studies were randomized controlled trials; the remainder were retrospective or prospective cohort studies.

Donor age ranged from 37 to 75 years (mean 55.4 ± 10.4 years) and recipient age from 45.5 to 61.5 years (mean 56.9 ± 3.8 years), indicating generally comparable baseline characteristics across studies. MELD scores ranged from 12 to 23 (mean 16.3 ± 3.3), reflecting moderate disease severity across the included populations.

Early Allograft Dysfunction (EAD)

**Figure 2:**
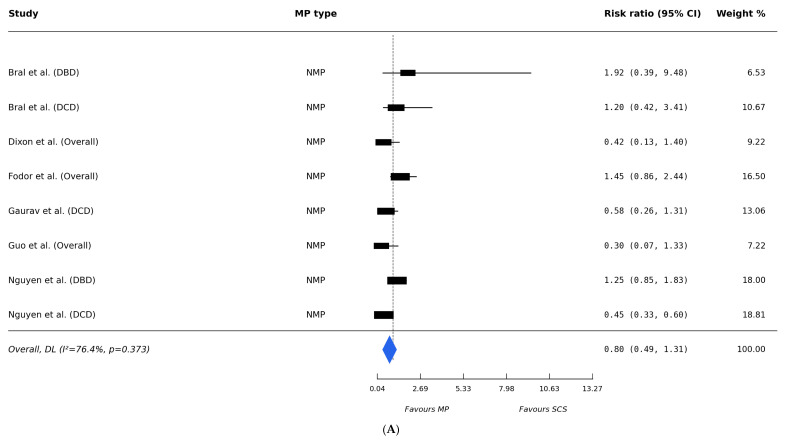

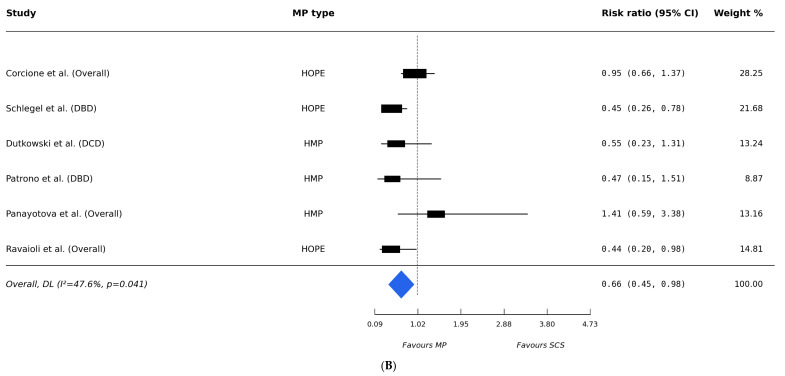
(**A**) Forest plot of early allograft dysfunction (EAD) in the NMP subgroup. Risk ratios with 95% confidence intervals are shown for each study and the pooled estimate. Studies included: Bral et al. [7], Dixon et al. [12], Fodor et al. [14], Gaurav et al. [15], Guo et al. [17], Nguyen et al. [21]. (**B**) Forest plot of early allograft dysfunction (EAD) in the HMP/HOPE subgroup, the diamond represents the pooled risk ratio estimate. Studies included: Corcione et al. [9], Schlegel et al. [10], Dutkowski et al. [13], Patrono et al. [23], Panayotova et al. [22], Ravaioli et al. [25]. DL = DerSimonian–Laird random-effects model.

Pre-specified subgroup analysis by perfusion modality was performed for EAD. In the NMP subgroup, no statistically significant reduction in EAD was observed compared with SCS (RR 0.80, 95% CI 0.49–1.31; *p* = 0.373), with substantial heterogeneity among included studies (I^2^ = 76.4%; Figure 2A).

By contrast, HMP/HOPE was associated with a significantly lower risk of EAD compared with SCS (RR 0.66, 95% CI 0.45–0.98; *p* = 0.041), with moderate heterogeneity (I^2^ = 47.6%; Figure 2B). Leave-one-out sensitivity analysis confirmed the robustness of this estimate across both subgroups.

Primary Non-Function (PNF)

**Figure 3:**
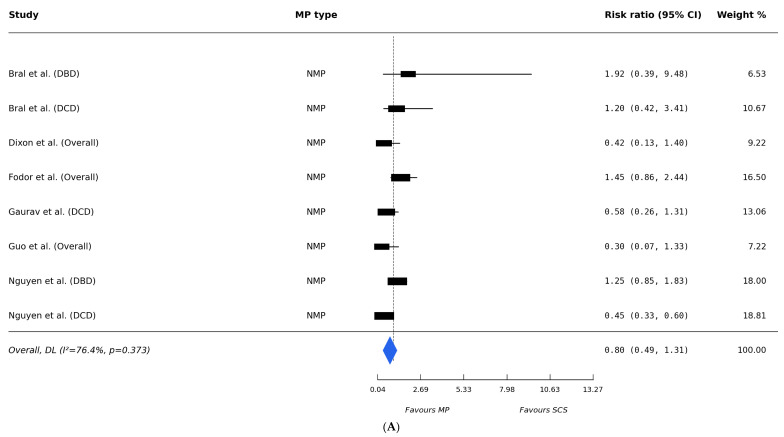

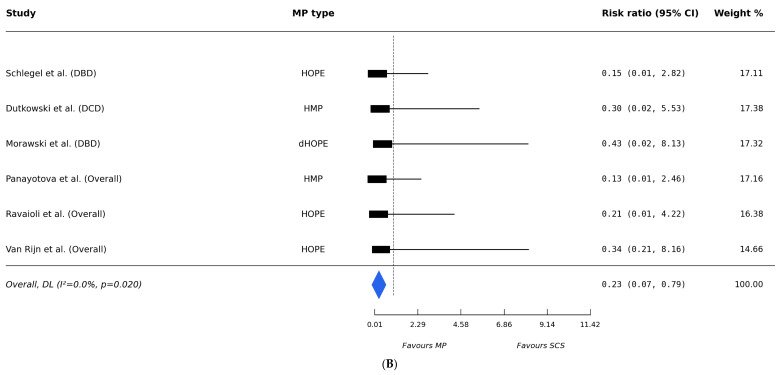
(**A**) Forest plot of primary non-function (PNF) in the NMP subgroup. Risk ratios with 95% confidence intervals are shown for each study and the pooled estimate. Studies included: Bral et al. [7], Dixon et al. [12], Fodor et al. [14], Gaurav et al. [15], Guo et al. [17], Nguyen et al. [21]. (**B**) Forest plot of primary non-function (PNF) in the HMP/HOPE subgroup. Studies included: Schlegel et al. [10], Dutkowski et al. [13], Morawski et al. [18], Panayotova et al. [22], Ravaioli et al. [25], Van Rijn et al. [27]. DL = DerSimonian–Laird random-effects model; the diamond represents the pooled risk ratio estimate.

Pre-specified subgroup analysis for PNF was similarly performed by perfusion modality. In the NMP subgroup, no statistically significant reduction in PNF was observed compared with SCS (RR 0.80, 95% CI 0.49–1.31; *p* = 0.373), with substantial heterogeneity (I^2^ = 76.4%; Figure 3A).

By contrast, HMP/HOPE was associated with a markedly and significantly lower risk of PNF compared with SCS (RR 0.23, 95% CI 0.07–0.79; *p* = 0.020), without significant heterogeneity (I^2^ = 0%; Figure 3B). Leave-one-out sensitivity analysis demonstrated stability of the pooled estimate across all included studies.

1-Year Graft Survival

**Figure 4:**
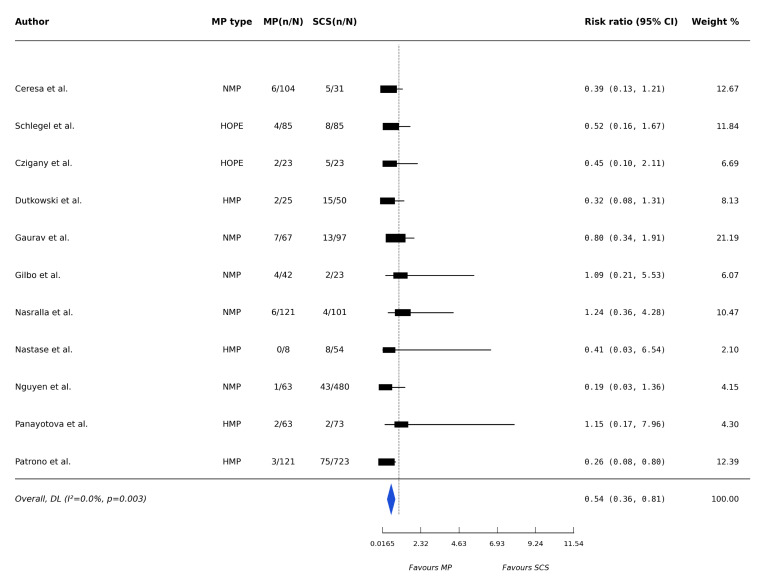
Forest plot of 1-year graft survival. Risk ratios with 95% confidence intervals are shown. Outcome is expressed as risk of graft loss (events/N). Studies included: Ceresa et al. [8], Schlegel et al. [10], Czigany et al. [11], Dutkowski et al. [13], Gaurav et al. [15], Gilbo et al. [16], Nasralla et al. [19], Nastase et al. [20], Nguyen et al. [21], Panayotova et al. [22], Patrono et al. [23]. I^2^ = 0% indicates negligible heterogeneity across included. The diamond represents the pooled risk ratio estimate.

The pooled analysis of 1-year graft survival demonstrated a significant and consistent benefit of machine perfusion over static cold storage, with a 46% reduction in the risk of graft loss (RR 0.54, 95% CI 0.36–0.81; *p* = 0.003). Notably, heterogeneity across studies was negligible (I^2^ = 0%; *p* = 0.681), indicating highly consistent results across all included studies and perfusion modalities (Figure 4).

Biliary Complications

The pooled analysis demonstrated no statistically significant difference in the incidence of biliary complications between machine perfusion and static cold storage (RR 0.82, 95% CI 0.60–1.10; *p* = 0.186), with moderate heterogeneity (I^2^ = 41.3%).

Hepatic Artery Thrombosis (HAT)

Machine perfusion was not associated with a statistically significant reduction in hepatic artery thrombosis compared with static cold storage (RR 0.74, 95% CI 0.36–1.51; *p* = 0.404), with minimal heterogeneity (I^2^ = 2.9%).

Liver Injury Markers

For AST, the overall pooled analysis demonstrated a statistically significant reduction in the MP group compared with SCS (SMD −3.04, 95% CI −5.27 to −0.81; *p* = 0.008); however, heterogeneity was extreme (I^2^ = 100%; *p* < 0.001). In sensitivity analysis excluding studies with extreme variance, the overall effect was no longer statistically significant (SMD −1.97, 95% CI −4.22 to 0.28; *p* = 0.086). In the HOPE subgroup, reduction in AST remained statistically significant (*p* = 0.014); no significant difference was observed in the NMP subgroup (*p* = 0.862). The test for subgroup differences was not statistically significant (*p* = 0.278).

**Figure 5:**
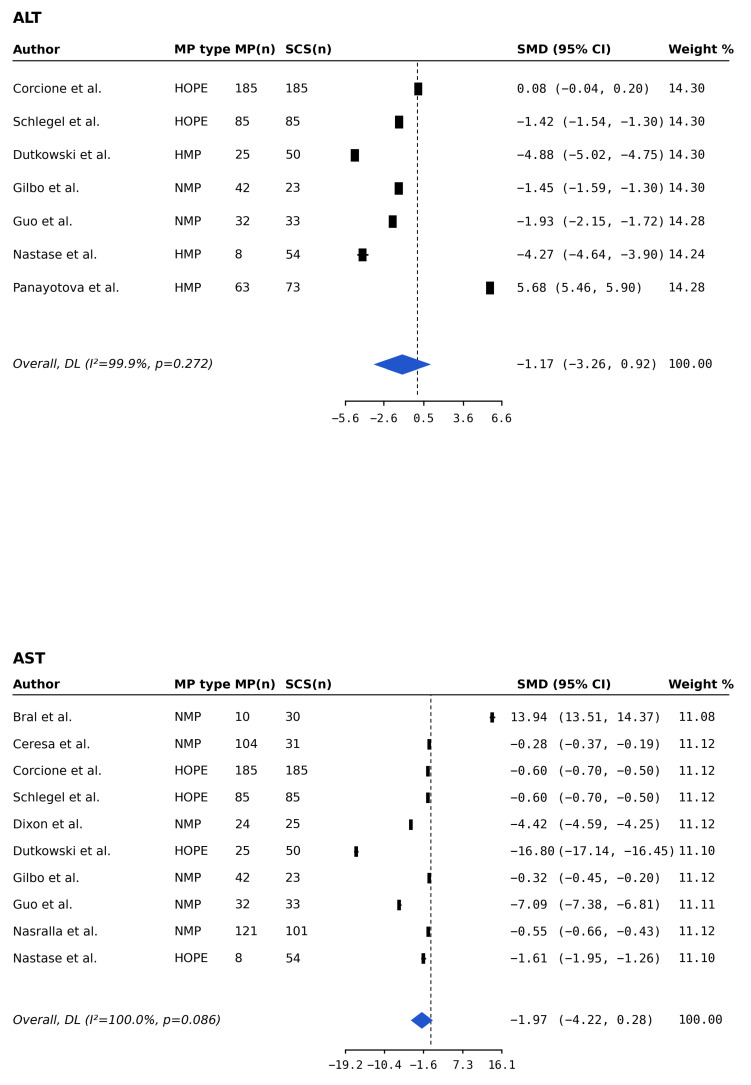
Forest plots of liver injury markers (ALT and AST). Forest plot of alanine aminotransferase levels. Studies included: Corcione et al. [9], Schlegel et al. [10], Dutkowski et al. [13], Gilbo et al. [16], Guo et al. [17], Nastase et al. [20], Panayotova et al. [22]. (AST) Forest plot of aspartate aminotransferase levels. Studies included: Bral et al. [7], Ceresa et al. [8], Corcione et al. [9], Schlegel et al. [10], Dixon et al. [12], Dutkowski et al. [13], Gilbo et al. [16], Guo et al. [17], Nasralla et al. [19], Nastase et al. [20]. SMD = standardized mean difference; DL = DerSimonian–Laird random-effects model; the diamond represents the pooled estimate.Standardized mean differences with 95% confidence intervals are shown. DL = DerSimonian–Laird random-effects model. Abbreviations: ALT—alanine aminotransferase; AST—aspartate aminotransferase; MP—machine perfusion; SCS—static cold storage; NMP—normothermic machine perfusion; HMP—hypothermic machine perfusion; HOPE—hypothermic oxygenated perfusion. The diamond represents the pooled risk ratio estimate.

For ALT, no statistically significant difference was observed between MP and SCS (SMD −1.17, 95% CI −3.26 to 0.92; *p* = 0.272), with extreme heterogeneity (I^2^ = 99.9%; *p* < 0.001; Figure 5). Given the extreme heterogeneity in transaminase analyses, these findings should be interpreted with caution; peak transaminase levels are influenced by multiple factors beyond preservation strategy, including total ischemia time, donor graft quality, intraoperative reperfusion technique, and surgical trauma.

Risk ratios with 95% confidence intervals are shown. Outcome is expressed as risk of graft loss (events/N). I^2^ = 0% indicates negligible heterogeneity across included studies.

## 4. Discussion

This systematic review and meta-analysis, incorporating 21 comparative studies and data from 3665 patients, demonstrates that machine perfusion is associated with clinically meaningful improvements in early graft function, a substantially reduced risk of primary non-function, and significantly better 1-year graft survival compared with static cold storage in adult liver transplantation. These findings are consistent across multiple centers, donor types, and perfusion modalities, and are supported by a robust body of evidence including six randomized controlled trials.

Organ shortage remains one of the principal challenges in liver transplantation, driving increasing utilization of ECD grafts and DCD organs that are inherently more vulnerable to preservation-related injury [28]. Static cold storage, while simple and widely applicable, does not halt metabolic processes entirely, and ischemia–reperfusion injury remains a major determinant of early graft dysfunction [29,30]. ECD grafts are particularly susceptible, exhibiting impaired sinusoidal microcirculation and reduced tolerance to oxidative stress [31].

From a mechanistic standpoint, the benefits of machine perfusion are biologically plausible. Continuous perfusion and oxygen delivery during preservation restores mitochondrial respiration, replenishes cellular energy stores, improves sinusoidal endothelial integrity, and attenuates the inflammatory cascade associated with reperfusion [32]. These processes are consistent with the observed reductions in EAD and PNF, both of which are direct clinical manifestations of ischemia–reperfusion injury [33].

Several prior meta-analyses have examined the role of machine perfusion in liver transplantation, providing important context for the present findings. Jaber et al. [34] conducted a systematic review, pairwise, and network meta-analysis restricted exclusively to randomized controlled trials, incorporating multiple perfusion modalities including HOPE, dual-HOPE, NMP, NMP-ischemia-free liver transplantation, and controlled oxygenated rewarming. While that analysis offers the advantage of indirect comparisons across modalities via network methodology, the present review broadens the evidence base by incorporating both RCT and high-quality observational studies, thereby reflecting real-world clinical practice and increasing the number of patients available for pooled analysis [34]. Tang et al. focused specifically on HOPE, pooling data from five RCTs and six matched studies (1000 patients) and demonstrated that HOPE reduced biliary complications, non-anastomotic strictures, EAD, acute rejection, and retransplantation rates, while improving 1-year graft survival compared to static cold storage [35].

A central and novel contribution of the present study is the modality-specific subgroup analysis. Hypothermic perfusion strategies, including HMP and HOPE, were associated with significant reductions in both EAD (RR 0.66) and PNF (RR 0.23), whereas NMP did not achieve statistical significance for these early graft function outcomes. These findings suggest that hypothermic and normothermic strategies exert distinct biological effects and should not be interpreted as a single homogeneous preservation category. Hypothermic perfusion primarily aims to restore aerobic mitochondrial metabolism while minimizing overall metabolic demand, which may be particularly effective in limiting the cellular energy depletion that underpins EAD and PNF. Normothermic perfusion, by contrast, more closely reproduces physiological conditions and offers the advantage of real-time viability assessment, but may not provide equivalent protection against the early biochemical manifestations of preservation injury in the included study populations.

Importantly, the 1-year graft survival benefit was consistent across both modalities (I^2^ = 0%), suggesting that the downstream impact of machine perfusion on graft durability is shared across perfusion strategies, even when early biochemical benefits differ. This finding is particularly clinically relevant in an era of expanding marginal graft utilization, where early preservation-related injury may have lasting consequences for long-term graft function.

The absence of a significant reduction in biliary complications or hepatic artery thrombosis is biologically plausible. Both outcomes are inherently multifactorial, influenced not only by the preservation strategy but also by surgical technique, vascular anatomy, donor arterial quality, and recipient-related factors. HAT is a major determinant of post-transplant biliary morbidity, as compromised arterial inflow leads to ischemic biliary injury predisposing to stricture and leakage [36]. The comparable rates of both complications between MP and SCS groups suggest that machine perfusion, while improving parenchymal preservation, may not fully mitigate complications driven predominantly by technical and vascular factors.

The extreme heterogeneity observed in transaminase analyses (I^2^ = 100%) reflects the inherent limitations of these biomarkers as endpoints in this context. Peak AST and ALT levels are influenced by multiple perioperative variables beyond preservation strategy alone, including total ischemia duration, intraoperative manipulation, and reperfusion technique. The reversal of the AST effect in sensitivity analysis after exclusion of studies with extreme variance further underscores the unreliability of isolated transaminase values as a primary measure of preservation efficacy. Composite clinical endpoints such as EAD, PNF, and graft survival are more robust and clinically meaningful.

Several limitations of this analysis warrant acknowledgement. First, the included studies were heterogeneous in design, with the majority being retrospective cohort studies, which are susceptible to selection bias and unmeasured confounding. Despite subgroup stratification by perfusion modality, residual heterogeneity persisted for several outcomes, attributable to differences in donor type (DBD versus DCD), graft quality, perfusion protocols, preservation duration, and outcome definitions. Second, long-term survival data beyond one year remain sparse across the included studies. Third, cost-effectiveness data were not available for pooled analysis. Fourth, the use of EAD as a primary outcome may be limited by variable definitions across studies, though the Olthoff criteria were most commonly applied. Fifth donor and recipient demographic variables including age, sex, disease aetiology, MELD score at transplantation, and immunosuppression protocols were not uniformly reported across included studies and could not be extracted for subgroup analysis; individual patient data meta-analysis would be required to fully account for these sources of heterogeneity. Sixth, cold ischaemia time for SCS and total perfusion duration for MP were inconsistently reported across studies and were not extracted as primary variables; differences in preservation duration between modalities represent a potential confounding factor that warrants dedicated investigation in future studies. Seventh, publication bias could not be formally assessed owing to the limited number of studies per subgroup, which precludes reliable funnel plot interpretation.

Despite these limitations, the overall body of evidence supports machine perfusion as a safe and effective preservation strategy that improves key early and short-term outcomes after liver transplantation. The modality-specific findings suggest potential clinical implications: hypothermic strategies appear promising for reducing early graft dysfunction and primary non-function, and may be particularly relevant for high-risk ECD and DCD grafts, though dedicated subgroup analyses in these populations are needed before definitive recommendations can be made. Normothermic perfusion may offer complementary advantages through real-time viability assessment. Further high-quality multicenter randomized trials with standardized outcome definitions, longer follow-up, and direct head-to-head comparisons of perfusion modalities, and dedicated analyses in ECD and DCD populations are required to clarify optimal strategy selection, cost-effectiveness, and long-term outcomes.

Beyond organ preservation, machine perfusion opens several promising translational avenues. Normothermic machine perfusion has demonstrated technical compatibility with ex situ liver splitting, potentially enabling viability assessment and metabolic stabilisation of each graft segment prior to implantation—an approach that may expand the applicability of split-liver transplantation to marginal grafts that would otherwise be declined. Furthermore, the sustained hepatocellular viability maintained during normothermic perfusion creates a biologically favourable substrate for downstream applications including hepatocyte isolation and cell-based therapies. Preliminary experimental evidence supports the feasibility of isolating functionally viable hepatocytes from NMP-maintained livers deemed unsuitable for transplantation, with potential utility in bioartificial liver support systems and regenerative medicine. These emerging applications underscore that machine perfusion represents not merely a preservation modality but a dynamic ex vivo platform with broader implications for transplant science and cell therapy.

## 5. Conclusions

Machine perfusion represents a significant advance in graft preservation for liver transplantation. Compared with static cold storage, it is associated with improved early graft function, substantially reduced primary non-function, and significantly better 1-year graft survival, with the benefit for early graft outcomes driven primarily by hypothermic perfusion strategies. Its role in reducing biliary complications and hepatic artery thrombosis remains uncertain. These modality-specific findings provide actionable evidence for clinical decision-making in preservation strategy selection. Future well-designed multicenter studies are needed to optimize modality selection, evaluate cost-effectiveness, and establish long-term outcomes.

## Figures and Tables

**Table 1:** Characteristics of included studies.

Study, Year	Country	MP Type	Patients	Donor Type	Study Design	ROB
M.Bral et al., 2017 [7]	Canada	NMP	40	DCD/DBD	Single-center/retrospective	Moderate
Ceresa et al., 2019 [8]	UK	NMP	135	DCD/DBD	Multi-center/prospective	Moderate
Corcione et al., 2025 [9]	Italy	HOPE	370	DBD	Single-center/retrospective	Low
Schlegel et al., 2023 [10]	Switzerland	HOPE	170	DBD	Multi/RCT	Moderate
Czigany et al., 2017 [11]	Germany	HOPE	46	ECD	Multi/RCT	Low
Dixon et al., 2023 [12]	USA	NMP	49	DCD/DBD	Single-center/retrospective	Low
Dutkowski et al., 2015 [13]	Switzerland	HMP	75	DCD/DBD	Multi-center/retrospective	Moderate
Fodor et al., 2021 [14]	Austria	NMP	118	DCD/DBD	Single-center/retrospective	Low
Gaurav et al., 2022 [15]	UK	NMP	164	DCD	Single-center/retrospective	Low
Gilbo et al., 2023 [16]	Belgium	NMP	65	DCD/DBD	Multi-center/retrospective	Moderate
Guo et al., 2023 [17]	China	NMP	65	ECD	Single-center/retrospective	Low
Morawski et al., 2024 [18]	Poland	dHOPE	104	DBD	Single-center/retrospective	Low
Nasralla et al., 2018 [19]	UK	NMP	222	DCD/DBD	Multi/RCT	Low
Nastase et al., 2025 [20]	Switzerland	HMP	62	DBD	Single-center/retrospective	Moderate
Nguyen et al., 2025 [21]	USA	NMP	543	DCD/DBD	Single-center/retrospective	Low
Panayotova et al., 2024 [22]	USA	HMP	136	DCD/DBD	Multi/RCT	Low
Patrono et al., 2021 [23]	Italy	dHOPE	844	DBD	Single-center/retrospective	Low
Pereyra et al., 2024 [24]	Austria	HOPE	247	DBD	Single-center/retrospective	Low
Ravaioli et al., 2022 [25]	Italy	HOPE	110	ECD	Single-center/prospective	Low
Selzner et al., 2016 [26]	Canada	NMP	40	DCD/DBD	Multi/RCT	Moderate
Rijn et al., 2025 [27]	Netherlands	HOPE	156	DCD	Single-center/retrospective	Low

NMP—normothermic machine perfusion; HMP—hypothermic machine perfusion; dHOPE—dual hypothermic machine perfusion; DCD—donor after circulatory death; DBD—donor after brain death; ECD—extended criteria donor; UK—United Kingdom; USA—United States of America; RCT—randomized controlled trial; ROB-risk of bias.

## Data Availability

Data supporting the findings of this review are available from the corresponding author upon request.

## Supplementary Materials

The following supporting information can be downloaded at: https://www.mdpi.com/article/10.3390/jcm15155950/s1. Reference [37] is cited in Supplementary Materials.

## Abbreviations

ALT—alanine aminotransferase; AST—aspartate aminotransferase; BCs—biliary complications; CI—confidence interval; DBD—donation after brain death; DCD—donation after circulatory death; dHOPE—dual hypothermic oxygenated perfusion; EAD—early allograft dysfunction; ECD—extended criteria donor; HAT—hepatic artery thrombosis; HMP—hypothermic machine perfusion; HOPE—hypothermic oxygenated perfusion; MELD—Model for End-Stage Liver Disease; MP—machine perfusion; NMP—normothermic machine perfusion; NOS—Newcastle-Ottawa Scale; PNF—primary non-function; PRISMA—Preferred Reporting Items for Systematic Reviews and Meta-Analyses; RCT—randomized controlled trial; ROB—risk of bias; RR—risk ratio; SCS—static cold storage; SMD—standardized mean difference.
